# Supplementary material for: Genome-Wide Association Study of Glucocerebrosidase Activity Modifiers
Source: Mol Neurobiol. 2025 Apr 29;62(9):11560–71. doi: 10.1007/s12035-025-04996-1 (PMC12367946; doi:10.1007/s12035-025-04996-1)
Supplement: Supplementary file 1 — Supplementary file1 (DOCX 9504 KB) [file 12035_2025_4996_MOESM1_ESM.docx]

**Supplementary Figures**
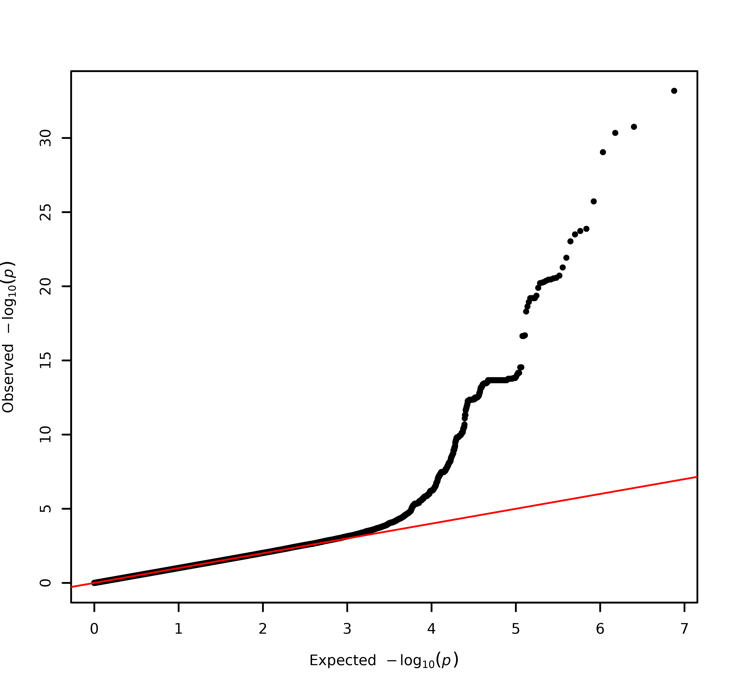

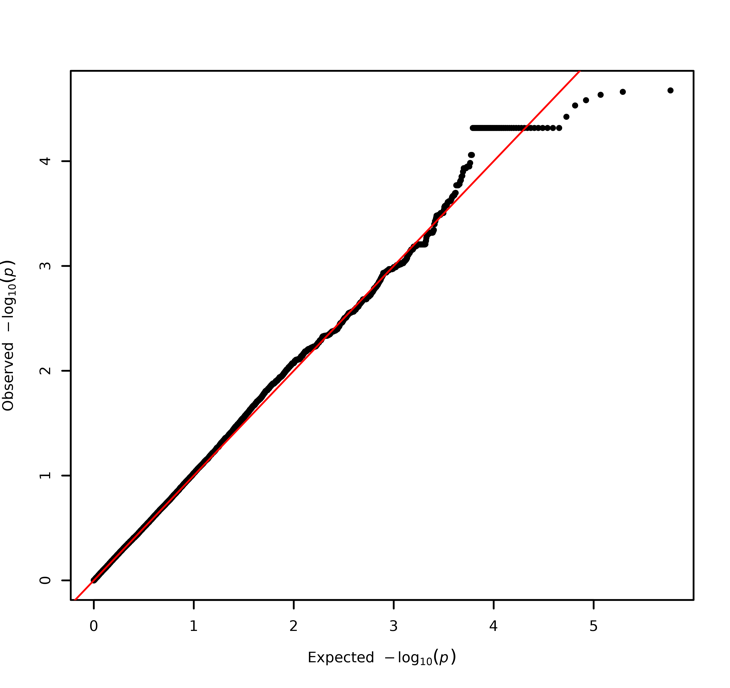


**a**

**b**

**Supplementary Figure 1.** QQ plots of linear regression p-values for a) the Columbia cohort with adjustments for age, sex, disease status, Ashkenazi Jewish status, *LRRK2* p.G2019S, ASM activity, GAA activity, GLA activity, GALC activity, and the top 10 PCs, b) the PPMI cohort with adjustments for age, sex, disease status, *LRRK2* G2019S genotype, ASM activity, GAA activity, GLA activity, GALC activity, white blood cell count, and the top 10 PCs


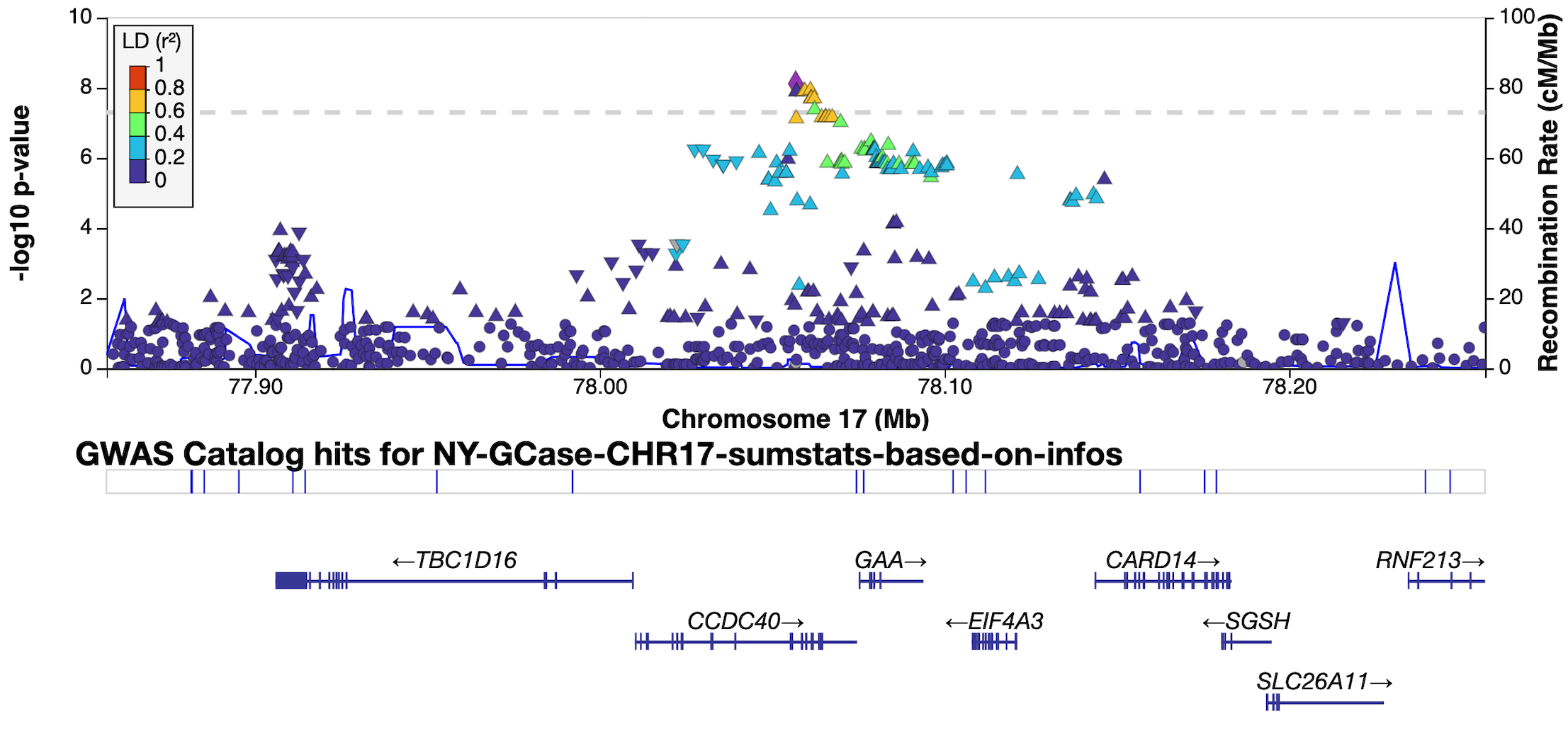


**Supplementary Figure 2**. LocusZoom Manhattan plot of the *GAA* locus from the analysis of the Columbia cohort. Color is described in the legend and based on linkage disequilibrium (R^2^).

**
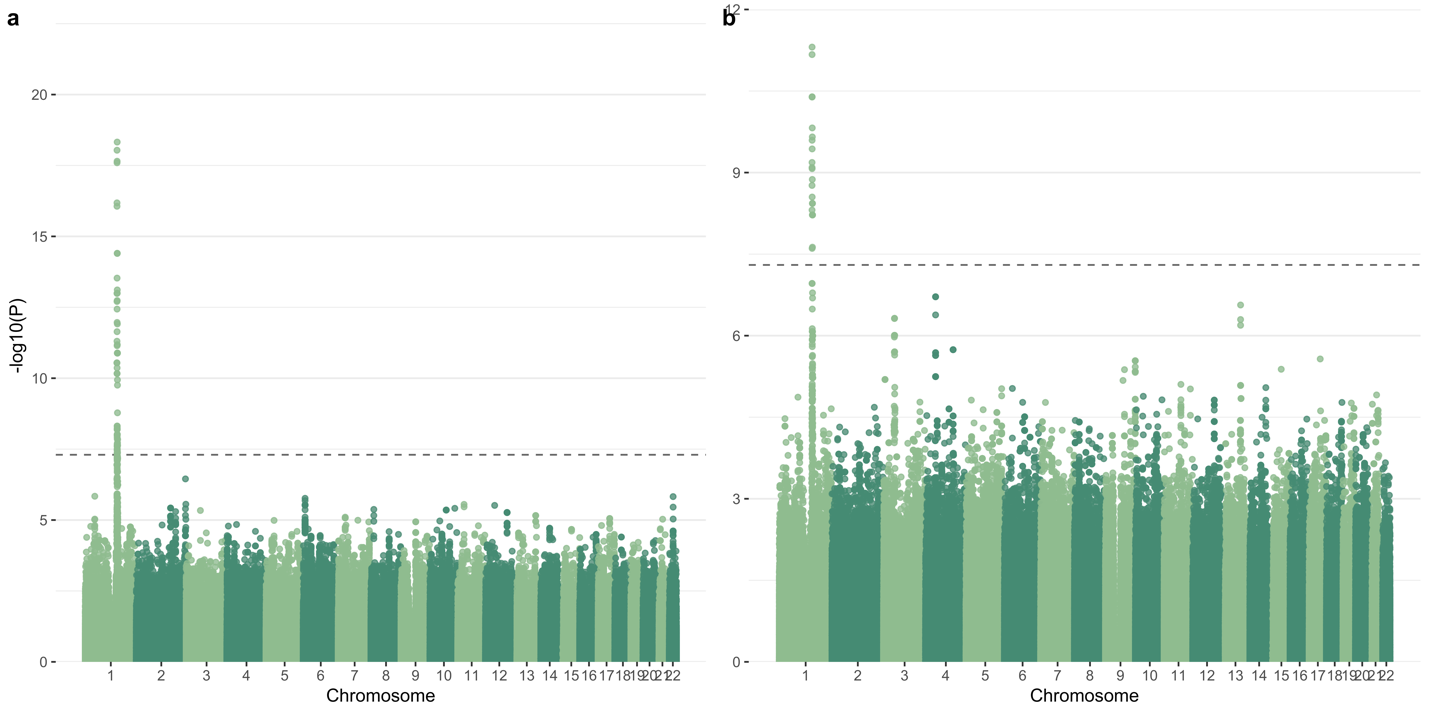
Supplementary Figure 3**. Manhattan plot of log adjusted p-values at each genomic position for the Columbia discovery cohort in a) PD cases and b) healthy controls with adjustments for age, sex, Ashkenazi Jewish status, *LRRK2* p.G2019S, ASM activity, GAA activity, GLA activity, GALC activity, and the top 10 PCs.


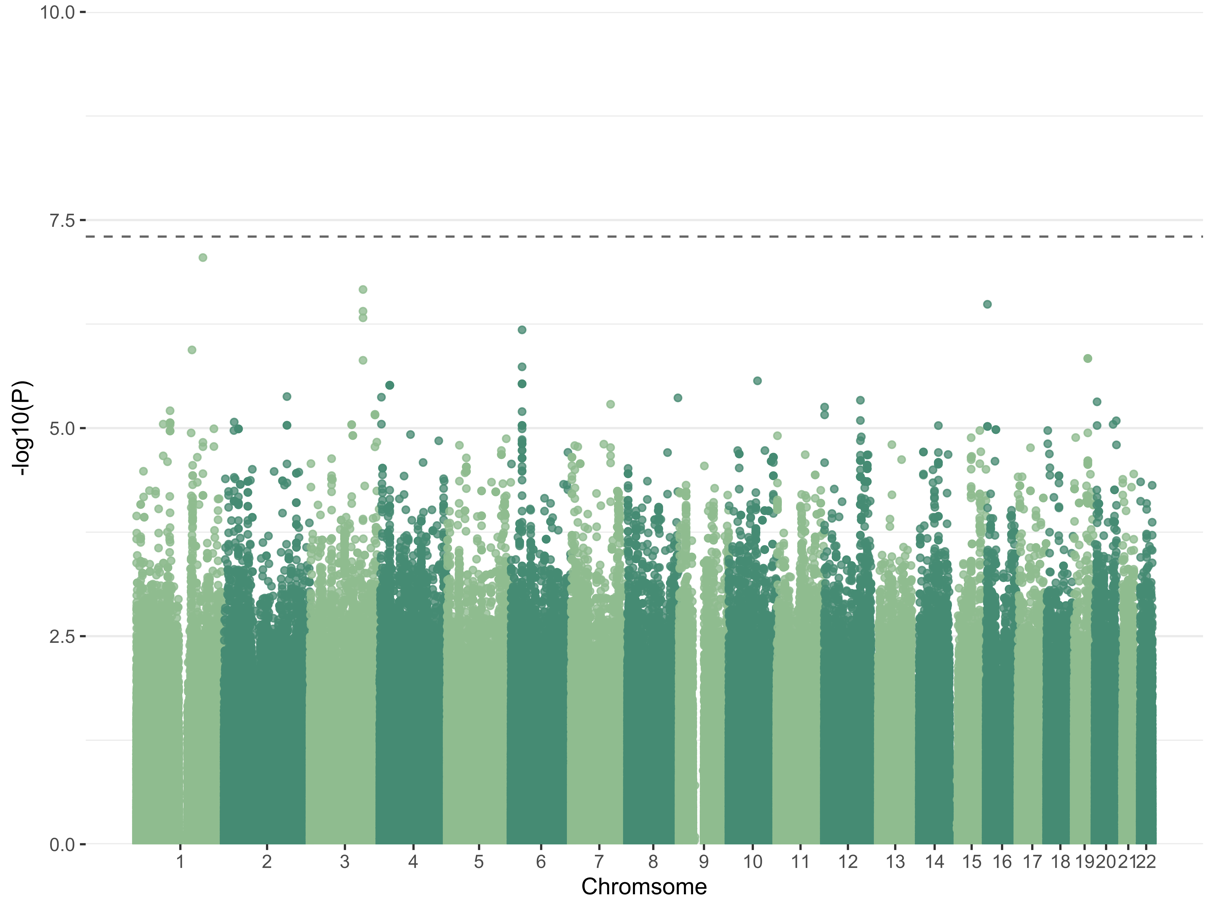


**Supplementary Figure 4**. Manhattan plot of log adjusted p-values at each genomic position for the meta-analysis of Columbia and PPMI cohorts using age, sex, disease status, *GBA1* p.N370S, p.E326K, and p.T369M genotype status, and 10 PCs.

**
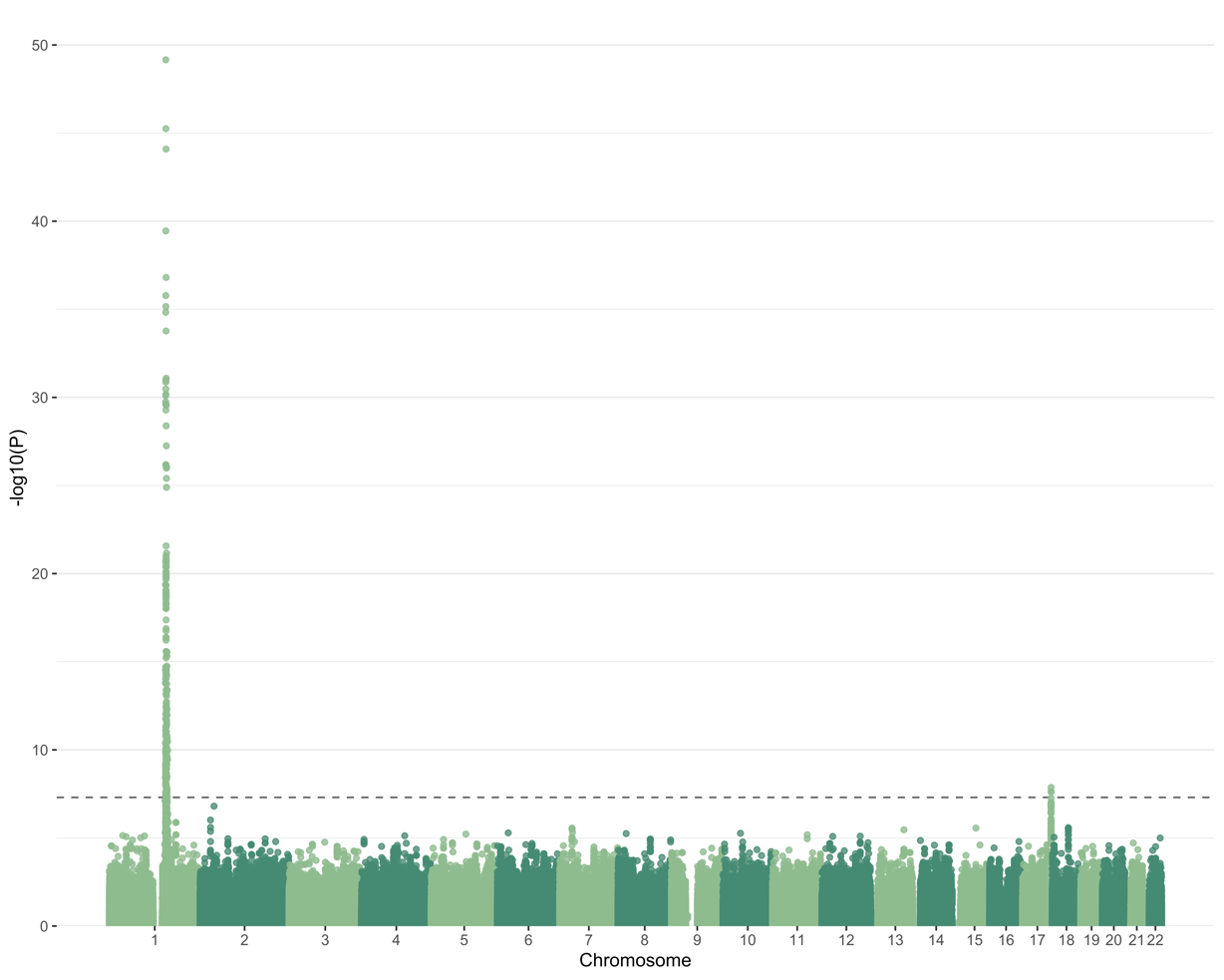
Supplementary Figure 5**. Manhattan plot of log adjusted p-values at each genomic position for the meta-analysis of Columbia and PPMI cohorts using main analysis covariates and including GCase activity outliers.
